# Radiolunate Fusion After Pyogenic Arthritis Caused by Pinning for Scapholunate Dissociation

**DOI:** 10.7759/cureus.71417

**Published:** 2024-10-14

**Authors:** Takeshi Ogawa, Sho Iwabuchi, Yuichi Yoshii

**Affiliations:** 1 Department of Orthopedic Surgery, National Hospital Organization, Mito Medical Center, Ibaraki, JPN; 2 Department of Orthopaedic Surgery and Sports Medicine, Mito Kyodo General Hospital, Ibaraki, JPN; 3 Department of Orthopaedic Surgery, Tokyo Medical University Ibaraki Medical Center, Ibaraki, JPN

**Keywords:** osteomyelitis, percutaneous pinning, pyogenic arthritis, radiolunate fusion, scapholunate dissociation

## Abstract

Infections associated with percutaneous pinning of the fingers occur in a certain percentage of cases; however, osteomyelitis rarely leads to more severe suppurative arthritis of the hand. A 26-year-old woman presented with scapholunate dissociation and underwent percutaneous pinning. Although some signs of infection were found around the pin site at five weeks, the pins were removed seven weeks postoperatively. At 10 weeks, she experienced severe wrist pain, with radiography, computed tomography, and magnetic resonance imaging confirming distractive changes in the lunate, radius, and pyogenic arthritis. Consequently, the infection was controlled; however, it led to radiolunate fusion. A case of percutaneous pinning followed by lunate osteomyelitis or pyogenic wrist arthritis leading to radial lunate fusion has not been reported previously. The pin should be removed immediately if it becomes infected.

## Introduction

Scapholunate (SL) dissociations are found in 5% of wrist sprains [1], and SL ligament injuries are observed in 54.5% of fractures in the distal end of the radius [2]. Geissler described the arthroscopic classification (grades I-IV) and the management of carpal instability [3]. Patients with acute Geissler grade II or grade III injuries are most ideally suited for arthroscopically assisted reduction and pinning [3]. Garcia-Elias classified six stages of SL dissociation and recommended pinning as a treatment for stage I or II injuries [4]. However, infections associated with percutaneous pinning of the wrists are uncommon [5-7]; moreover, severe complications such as osteomyelitis progressing to septic arthritis of the wrist are exceedingly rare [8]. Botte et al. documented an 11% incidence of infection following percutaneous pinning, with 4% involving the carpal bones and osteomyelitis occurring in two out of 177 cases [9]. Septic arthritis of the wrist, although infrequent, constitutes a critical clinical condition, primarily involving the radiocarpal joint but potentially extending to the midcarpal and distal radioulnar joints. In advanced or neglected cases, the infection may spread into the carpal tunnel or adjacent deep soft tissues [10]. Moreover, Skeete et al. reported that septic arthritis in patients presenting with an acute, non-traumatic, swollen wrist in emergency settings had a prevalence of 5% in their cohort [11]. Their findings also highlighted that laboratory indices, including leukocyte count, erythrocyte sedimentation rate (ESR), and C-reactive protein (CRP), lacked sufficient specificity and sensitivity to definitively confirm or exclude septic arthritis of the wrist. We present a case of suppurative osteomyelitis and carpal arthritis following percutaneous pinning for scapholunate dissociation and further explore potential prophylactic strategies.

## Case presentation

**Figure 1:**
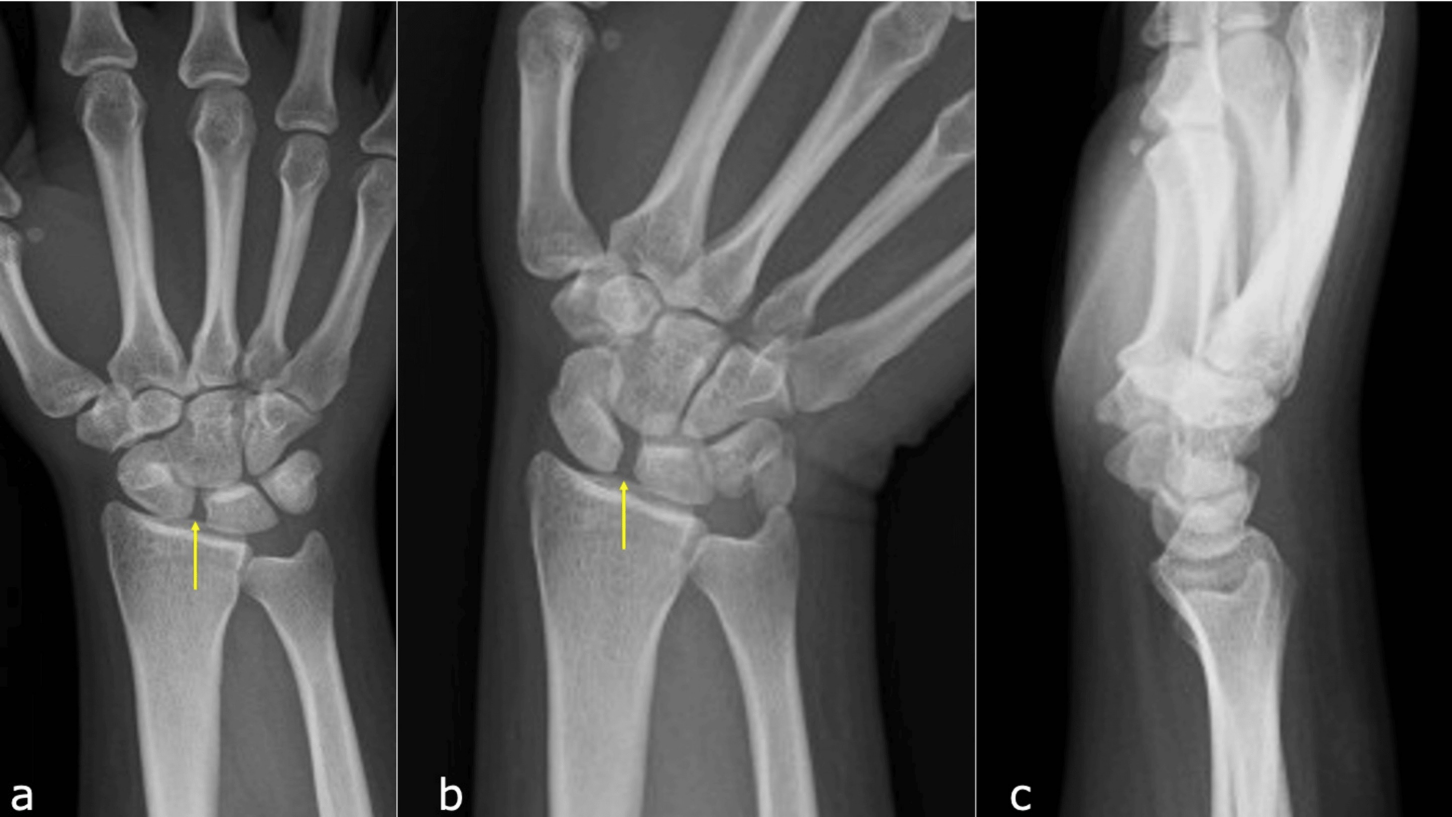
Initial X-ray at hospital admission. a: Anteroposterior (AP) view. b: AP view with ulnar deviation showing widening of the scaphoid-lunate interval (arrow). c: Lateral view, showing a radio-scaphoid angle of 40°.

A 26-year-old female dietitian sustained a compressive injury to her right hand, caught between a catering truck and a wall. Clinical examination revealed marked tenderness in the SL region, and radiographic imaging in the ulnar flexion position showed an increased SL interval (Figures 1a-1c).

**Figure 2:**
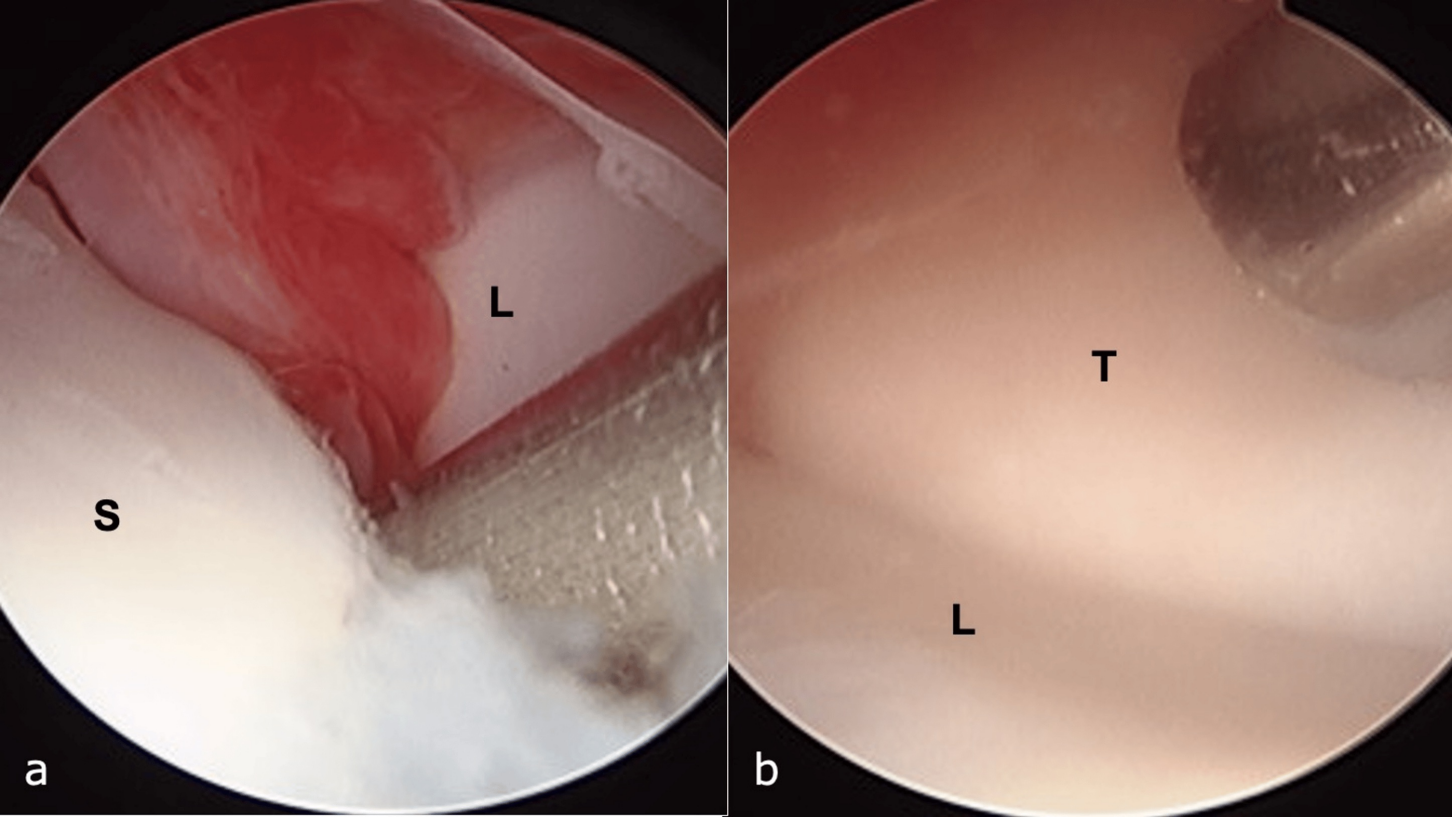
Arthroscopic findings. a: Scapholunate interval, classified as Geissler grade 3. b: Luno-triquetral interval classified as Geissler grade 1.

The patient was diagnosed with SL dissociation, classified as Garcia-Elias stage 2, and subsequently underwent surgery [4]. Under general anesthesia, arthroscopic evaluation of SL instability was performed using the Geissler classification [3], revealing grade 3 instability at the SL joint and grade 1 instability at the lunotriquetral joint (Figures 2a, 2b).

**Figure 3:**
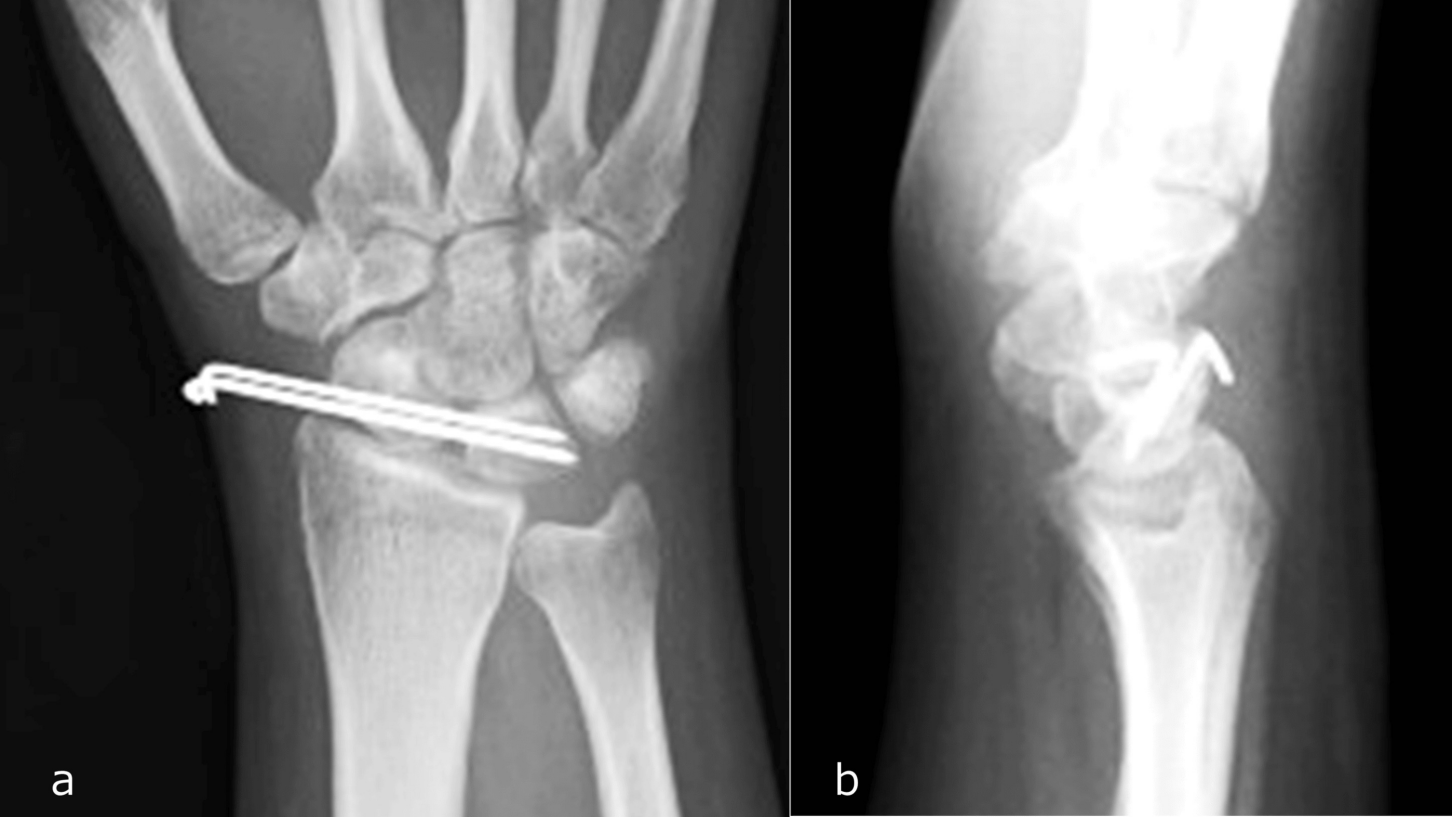
Postoperative X-ray following the initial surgery. a: Anteroposterior view. b: Lateral view.

Stabilization of the SL joint was achieved using two 1.5-mm Kirschner wires (K-wires) (Figures 3a, 3b), which were placed percutaneously and scheduled for removal after eight weeks.

**Figure 4:**
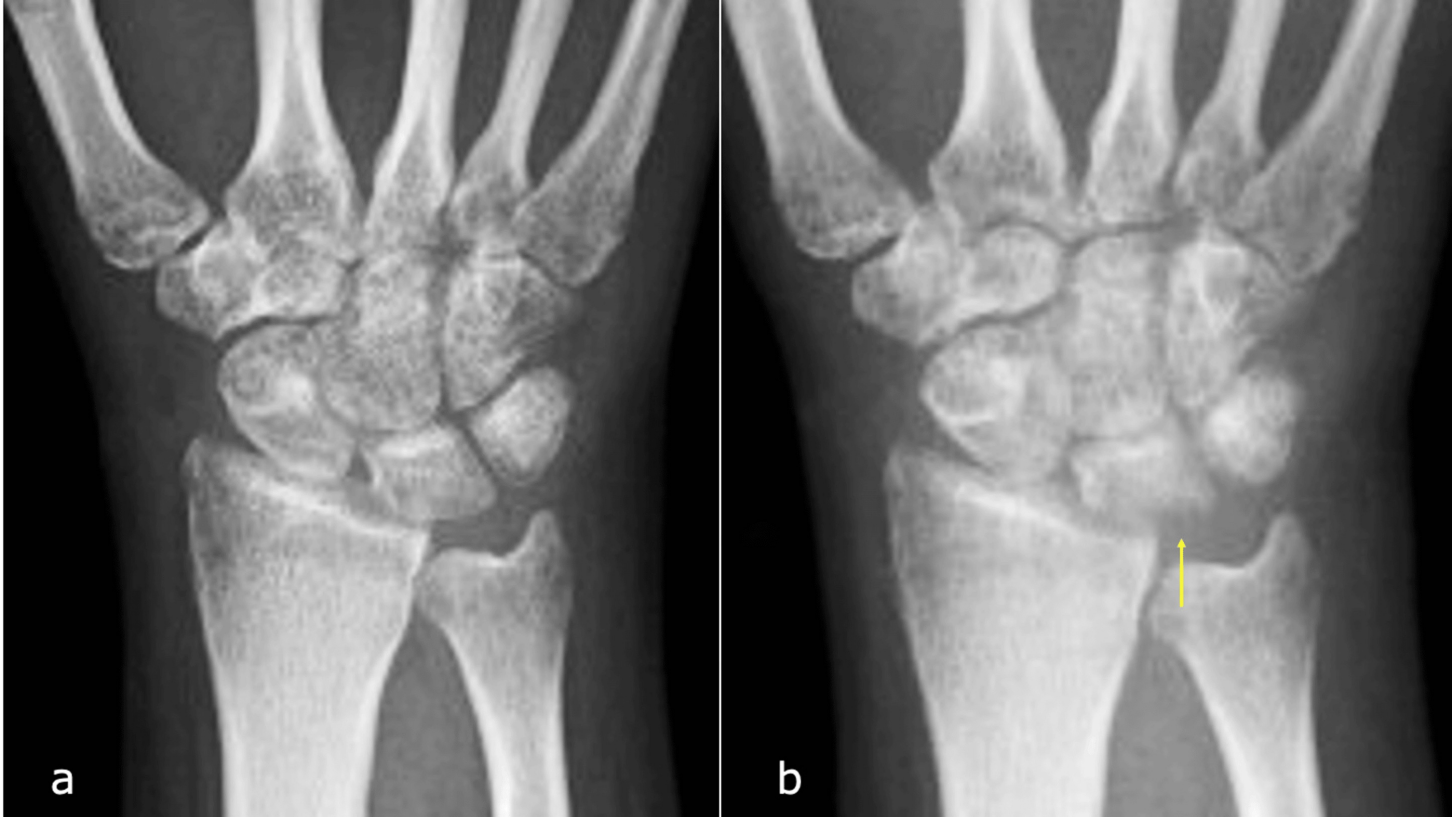
X-ray following Kirschner wire (K-wire) removal. a: Anteroposterior (AP) view immediately after K-wire removal. b: AP view three weeks post-K-wire removal. The irregularities of the proximal lunate articular surface can be seen (arrow).

Despite the patient reporting persistent postoperative pain, immobilization with a splint was continued. At five weeks postoperatively, the wrists were a little swollen overall; however, there was no redness or heat. Additionally, clear yellow exudate was observed at the K-wire entry site. The patient did not exhibit signs of systemic infection, and no worsening of symptoms occurred after showering, administration of nonsteroidal anti-inflammatory drugs (NSAIDs), or oral antibiotic therapy. The K-wire was removed at seven weeks postoperatively (Figure 4a).

**Figure 5:**
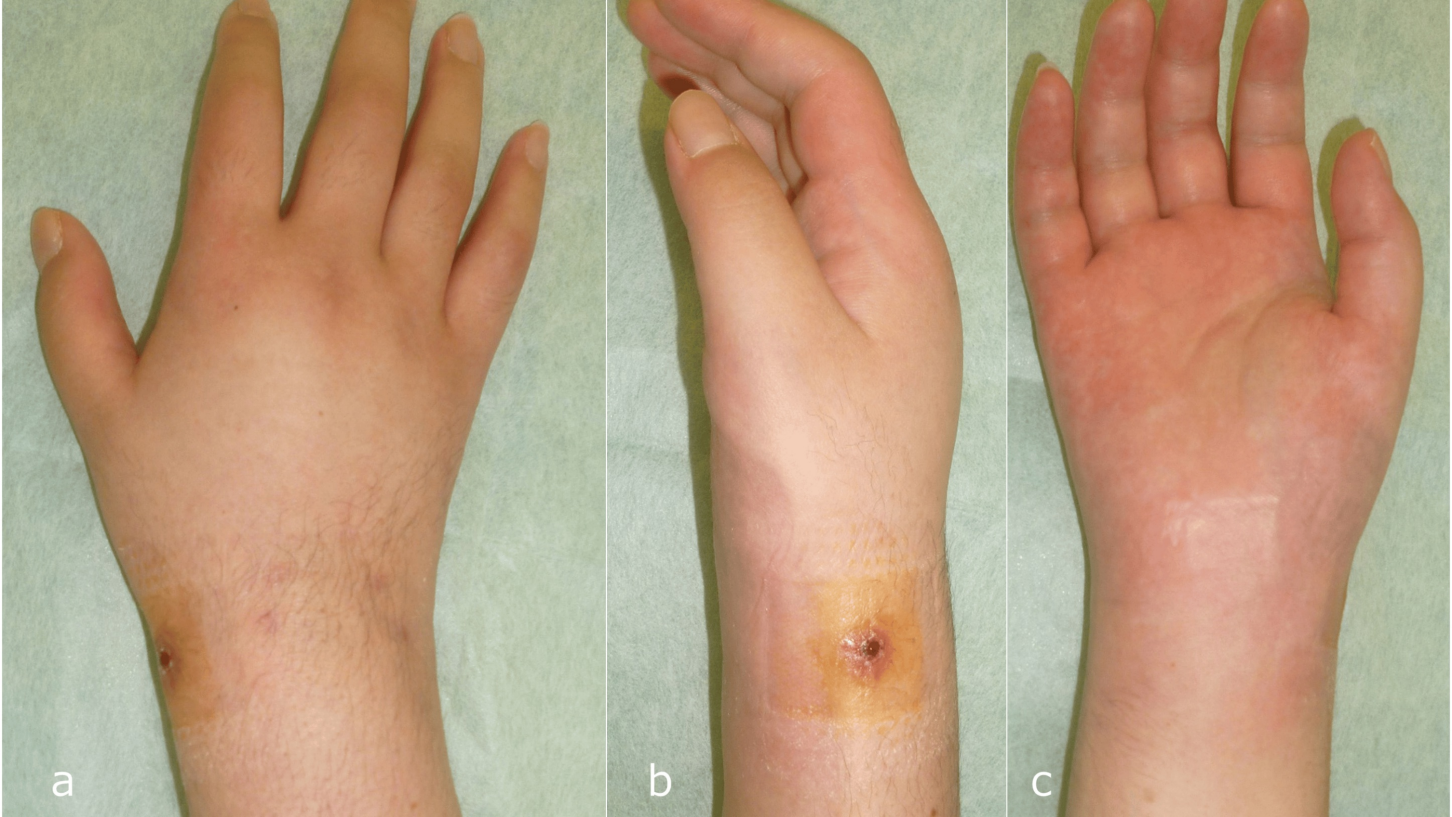
Clinical appearance three weeks after Kirschner wire removal. a: Dorsal view. b: Radial view. c: Volar view.

Three weeks post-removal, the patient experienced an exacerbation of swelling and increased warmth around the wrist (Figures 5a-5c).

**Figure 6:**
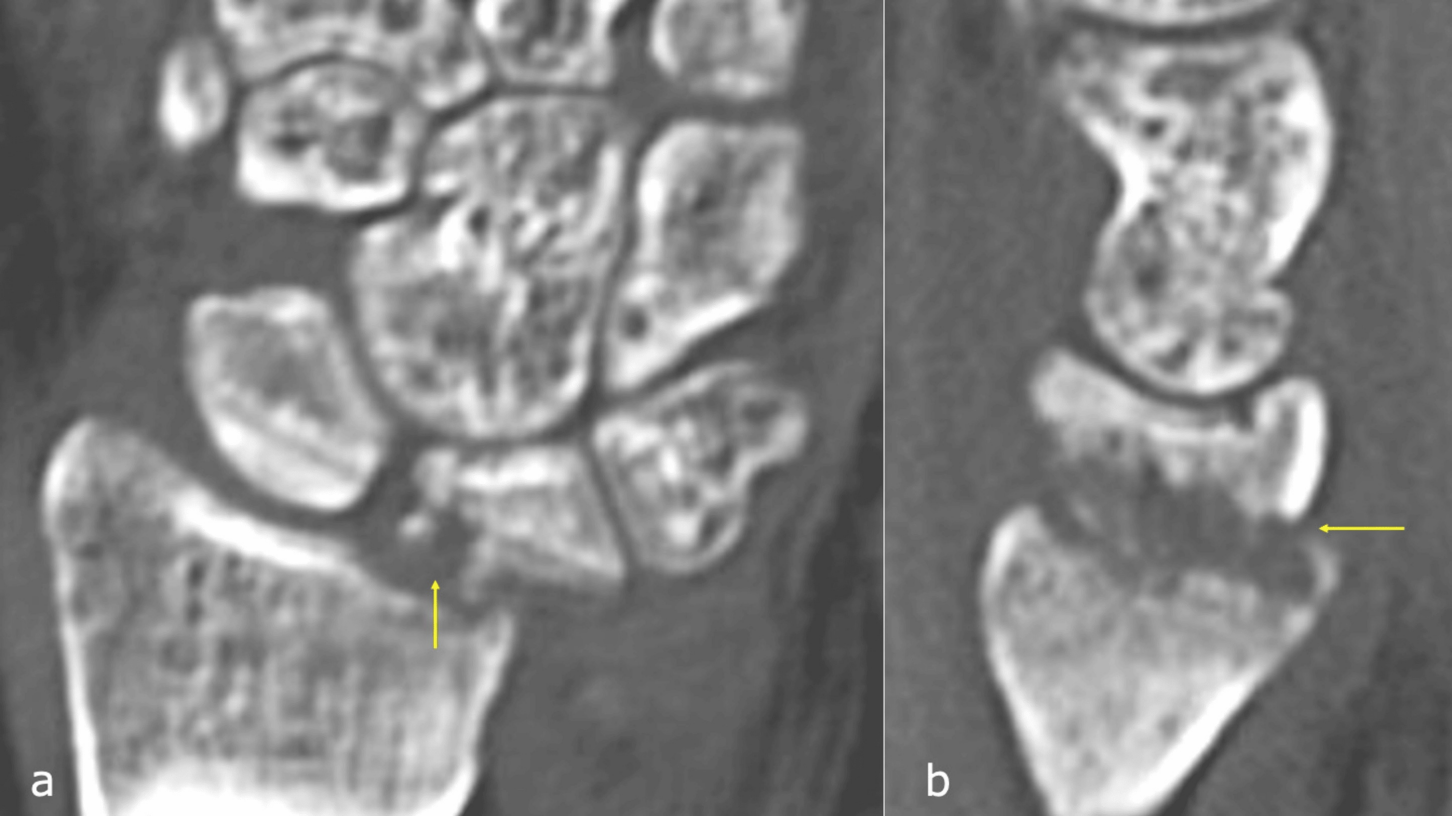
Computed tomography three weeks after Kirschner wire removal. a: Coronal view. b: Sagittal view. The lunate destruction and irregularity of the radial lunate fossa can be seen (arrow).

**Figure 7:**
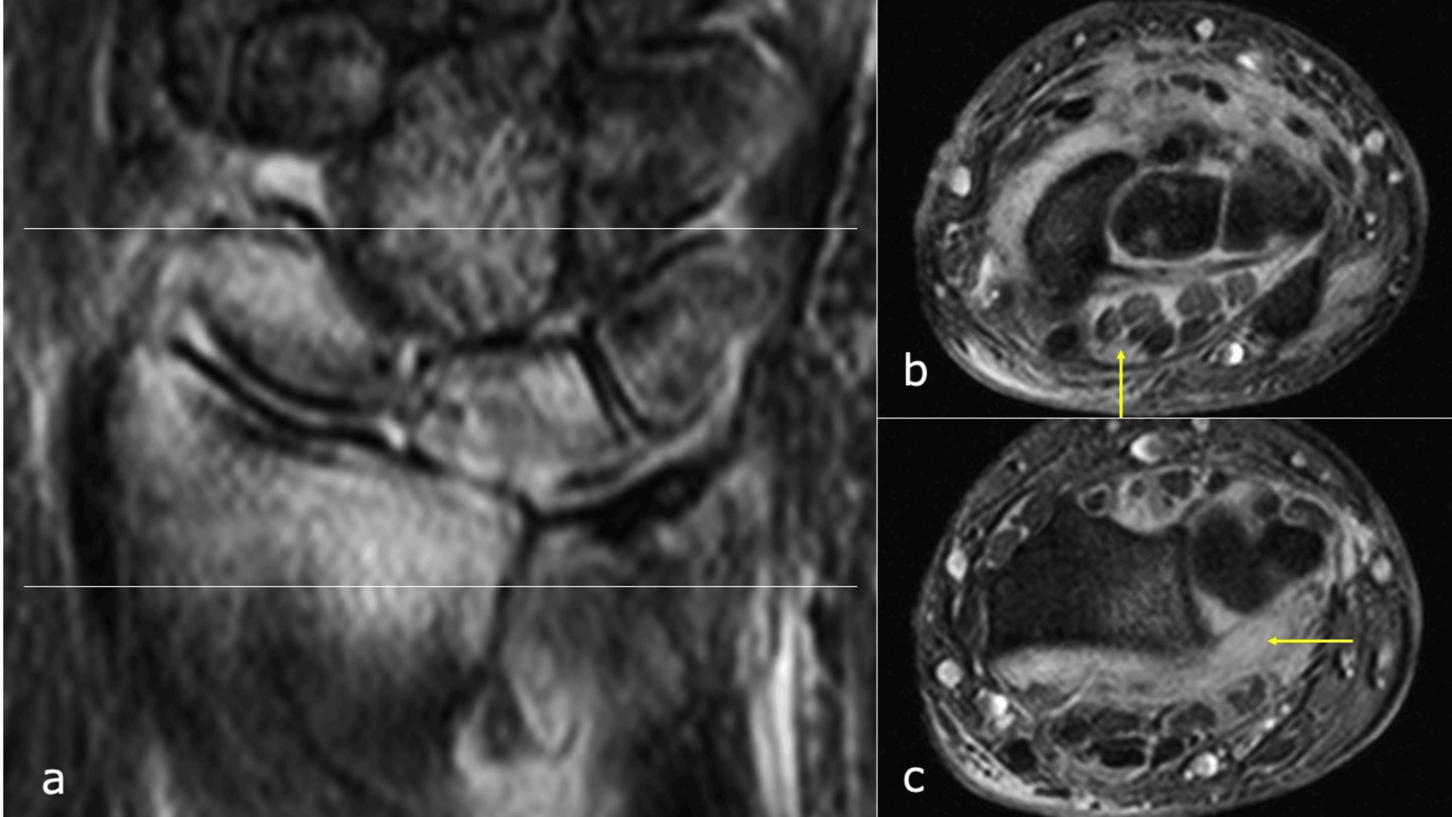
Magnetic resonance imaging three weeks after Kirschner wire removal. a: Coronal view. b: Axial view at the carpal tunnel level. c: Axial view at the distal radioulnar joint level. The inflammatory changes extending to the palmar aspect of the wrist joint and surrounding flexor tendons can be seen (arrow).

Radiographic evaluation revealed irregularities of the proximal lunate articular surface (Figure 4b). Laboratory investigations showed a white blood cell (WBC) count of 8.5 × 10^9^/L and a CRP level of 2.43 mg/dL. Computed tomography (CT) imaging showed osseous destruction of the lunate and irregularity of the radial lunate fossa (Figures 6a, 6b), whereas magnetic resonance imaging (MRI) revealed bone marrow edema in the lunate and inflammatory changes extending to the palmar aspect of the wrist joint and surrounding flexor tendons (Figures 7a-7c).

**Figure 8:**
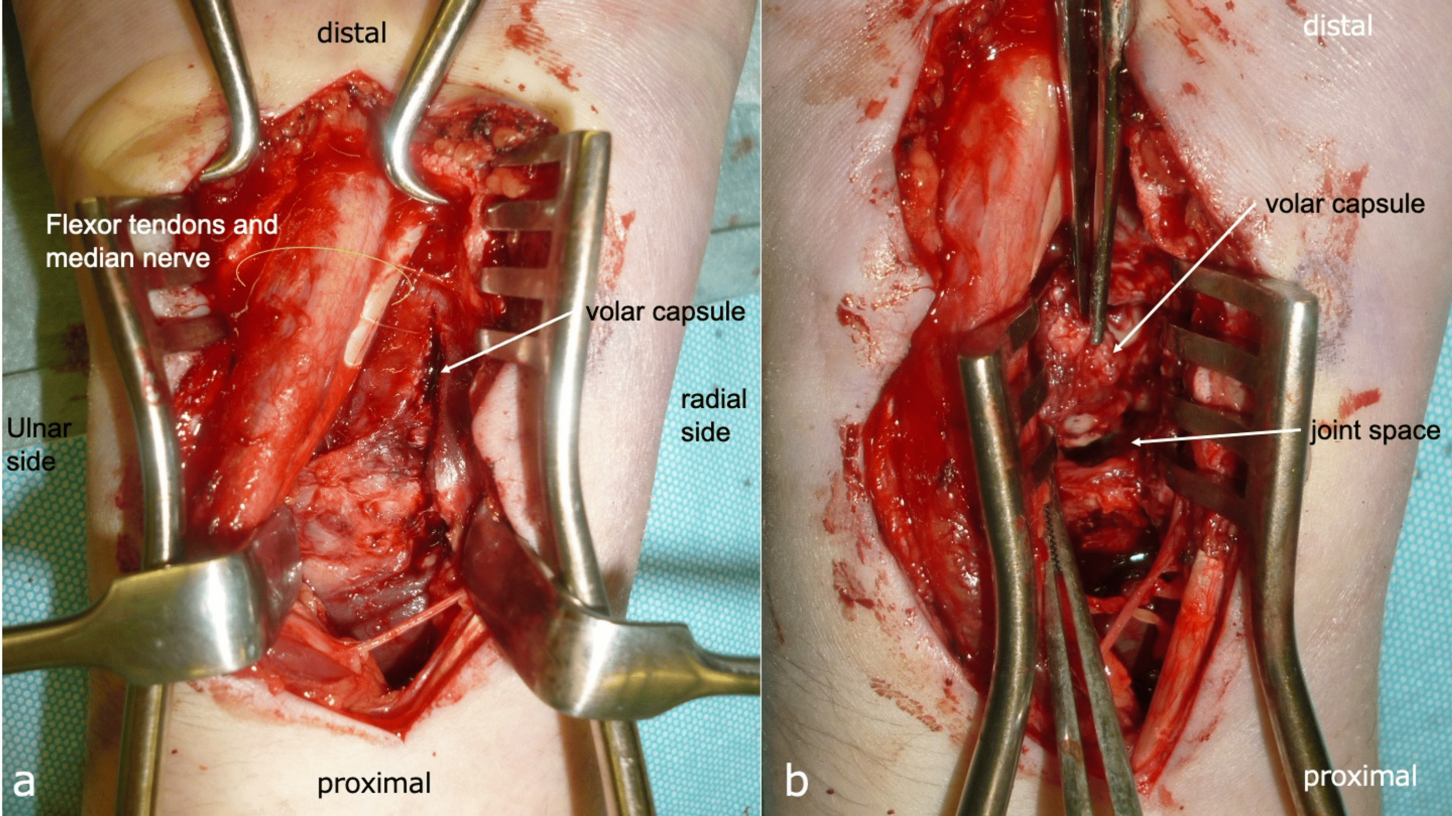
Findings from the second surgical procedure. a: Volar approach. b: Partial resection of the volar capsule.

**Figure 9:**
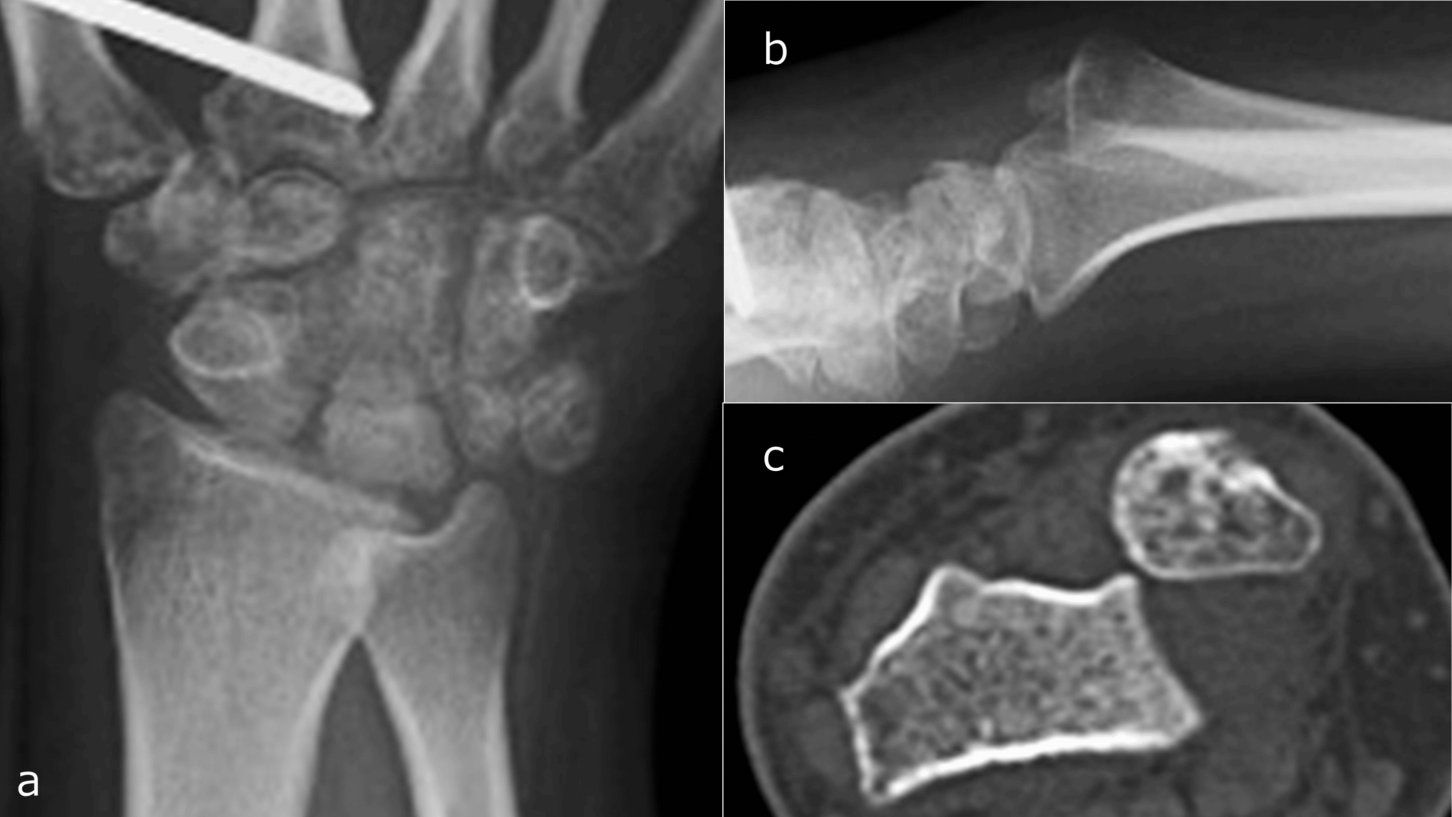
Post-second surgery imaging. a: X-ray anteroposterior view. b: X-ray lateral view. c: Computed tomography axial view.

Consequently, a diagnosis of lunate osteomyelitis with progression to pyogenic wrist arthritis was made, necessitating urgent surgical intervention. The patient underwent synovectomy of the inflamed synovial tissue around the flexor tendons via a palmar approach to the wrist joint. Extensive debridement and lavage of the wrist joint were performed (Figure 8a). Additionally, a portion of the infected and compromised palmar joint capsule of the distal radioulnar joint (DRUJ) was excised (Figure 8b), and external fixation was applied for wrist stabilization (Figure 9a). The palmar capsule of the DRUJ was left partially defective, resulting in dorsal dislocation of the ulnar head (Figures 9b, 9c).

**Figure 10:**
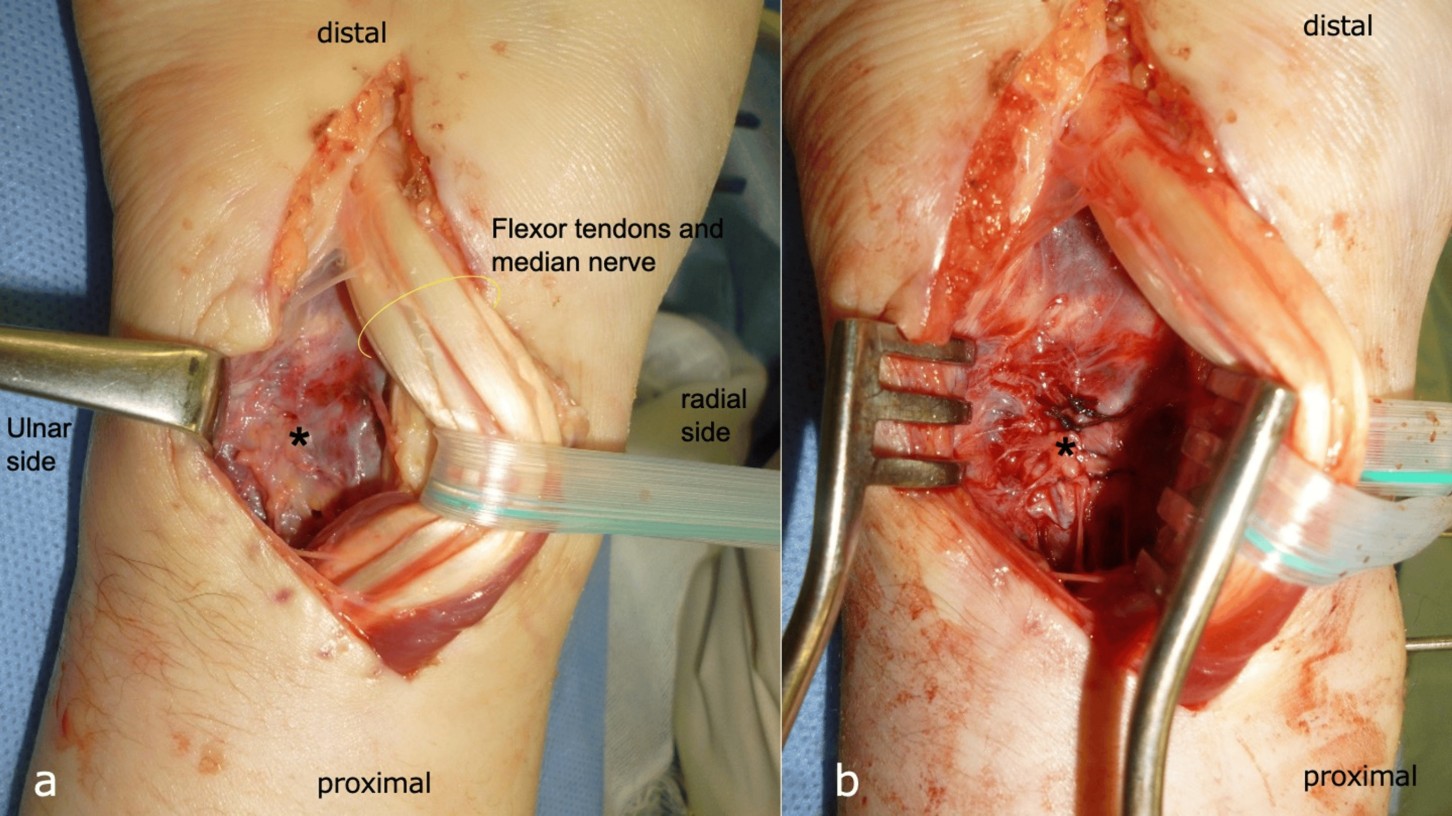
Findings from the third surgical procedure. a: Volar approach with minimal infectious tissue observed. b: Suturing of the volar capsule and distal radioulnar ligament. Asterisk (*) is the distal radioulnar joint.

Methicillin-sensitive *Staphylococcus aureus* (MSSA) was detected in the debridement tissues. Therefore, the patient received intravenous cefazolin (CEZ) 4 g/day, targeting MSSA. After three weeks, a second debridement and DRUJ stabilization were performed. The infection resolved, and DRUJ stability was reestablished by suturing the distal radioulnar ligament and joint capsule to the ulnar border of the radius (Figures 10a, 10b).

**Figure 11:**
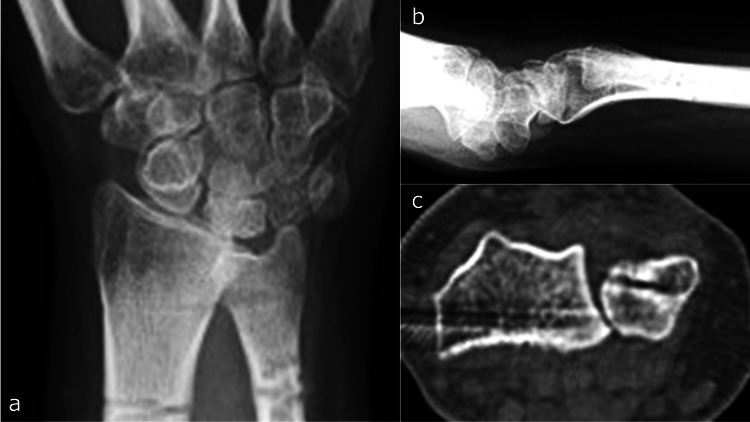
Imaging six months post-injury. a: X-ray anteroposterior view. b: X-ray lateral view. c: Computed tomography axial view.

Post-treatment laboratory results showed a WBC count of 4.6 × 10^9^/L and a CRP level of 0.11 mg/dL. Two K-wires were temporarily placed between the radius and ulna and removed after five weeks, followed by external fixation for two months. The patient received intravenous CEZ for six weeks, followed by oral cefaclor for one year. Six months postoperatively, there was no recurrence of infection, and DRUJ stability was maintained (Figures 11a-11c).

**Figure 12:**
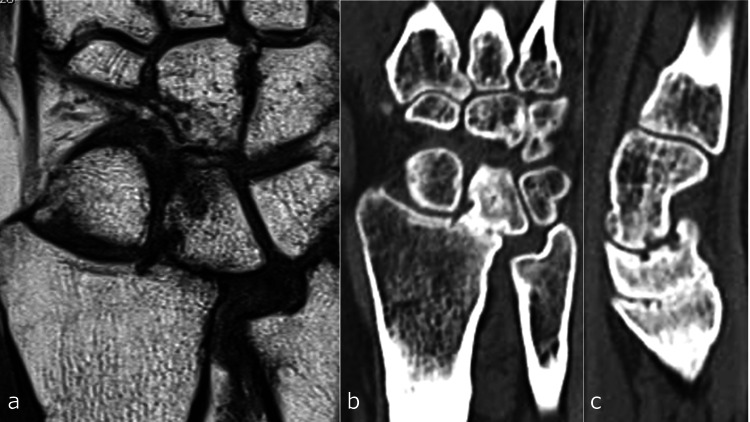
Imaging two years post-injury. a: Magnetic resonance imaging coronal view. b: Computed tomography (CT) coronal view. c: CT sagittal view.

However, the patient continued to experience pain and exhibited a significantly restricted range of motion (ROM): 20° dorsiflexion, 10° palmar flexion, 10° external rotation, and 45° internal rotation. Two years post-injury, MRI showed normalization of the marrow signal in the lunate and radius (Figure 12a); however, progressive cartilage degradation of the lunate and radial lunate fossa was noted, with CT imaging indicating a tendency toward lunate-radius fusion (Figures 12b, 12c).

**Figure 13:**
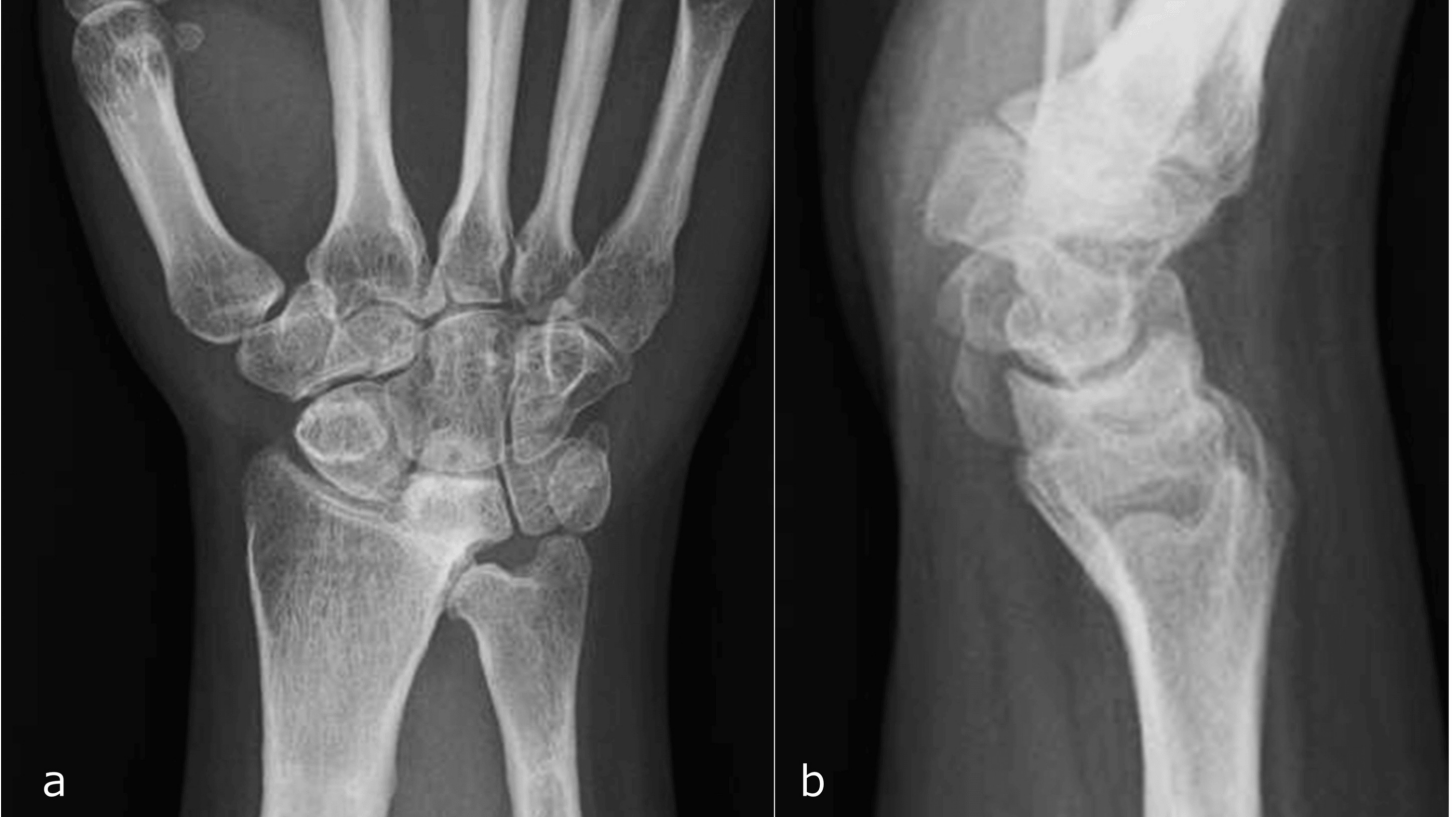
X-ray three years post-injury. a: Anteroposterior view. b. Lateral view.

Three years post-injury, complete fusion of the radius and lunate was observed (Figures 13a, 13b).

**Figure 14:**
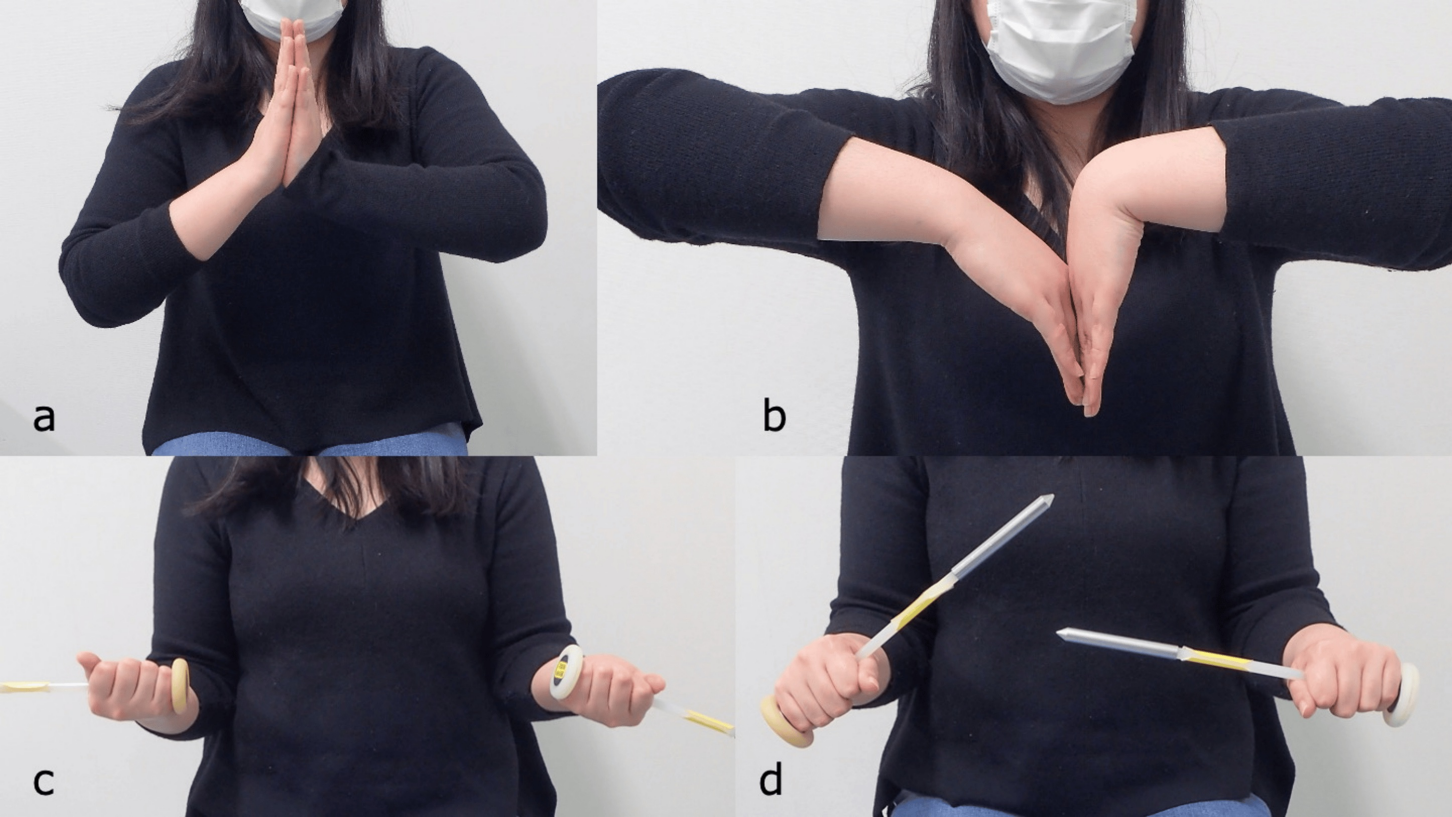
Wrist range of motion five years post-injury. a: Dorsiflexion. b: Volar flexion. c: Supination. d: Pronation.

At five years, there was no recurrence of infection, and the patient resumed her professional duties as a dietitian, with a grip strength of 20 kg (77% of normal). Nevertheless, her ROM remained limited to 40° dorsiflexion, 20° palmar flexion, 90° external rotation, and 60° internal rotation of the right wrist (Figures 14a-14d).

Her Mayo wrist score was 70 points, and her Disability of the Arm, Shoulder, and Hand (DASH) score was 6.25, with noted improvement in her subjective symptoms.

## Discussion

Botte et al. reported an 11% (45/422 pins) infection rate following percutaneous pinning for wrist fractures, with infection rates of 3% in middle phalanx cases, 2% in proximal phalanx cases, 1% in metacarpal cases, and 4% in carpal cases [9]. The mean duration for pin removal was 10 weeks (range = 1-21 weeks), with complications occurring in 22% of cases where pins were retained for more than 6.5 weeks. Osteomyelitis developed in two cases: one following a basal bone fracture with crush injury and another after periosteal dislocation of the lunate. In the latter case, an MSSA infection was identified 12 weeks postoperatively. Despite surgical debridement and six weeks of intravenous oxacillin, spontaneous fusion of the central carpal bones was observed two years later. These findings underscore the heightened susceptibility of the carpus to infection following percutaneous pinning in hand trauma, particularly with prolonged pin retention. In this study, although the K-wire was removed at seven weeks, the presence of exudate at five weeks suggests that the delay in removal likely contributed to the subsequent severe infection. Whether or not to bury the pin remains controversial; in this case, the pin could not be buried because of the location of the branches of the radial artery and nerve. Birman et al. recommend that in cases of postoperative infection or late presentation with purulent discharge, needle aspiration combined with empiric parenteral antibiotics should be initiated, and surgical intervention should be considered if there is no substantial improvement within 12 hours of aspiration [10]. Although serum biomarkers are routinely utilized in diagnosing septic arthritis, they lack definitive diagnostic value. For instance, a systematic review demonstrated that abnormalities in serum WBC, ESR, and CRP levels do not significantly alter the pretest probability of septic arthritis [12]. According to the study, a synovial fluid WBC count of ≥50,000 cells/μL indicates a high likelihood of septic arthritis, with counts ≥100,000 cells/μL further increasing this probability [12]. In another study of 202 patients with suspected septic arthritis, only 47% of cases with synovial WBC counts >50,000 cells/μL were confirmed as septic arthritis, whereas 77% of cases with counts >100,000 cells/μL had positive bacterial cultures. However, even the most reliable tests, such as synovial fluid Gram stain and culture, may not immediately confirm acute septic arthritis, as cultures often become positive only after several days [13]. Hunter et al. concluded that most cases of septic arthritis (62%) were successfully managed with a single surgical debridement [14]. Factors associated with a higher risk of requiring additional debridement include a history of inflammatory arthropathy, involvement of a large joint, a synovial fluid nucleated cell count >85.0 × 10^9^ cells/L, *S. aureus* infection, or diabetes.

In an 11-year study of 40 cases of pyogenic arthritis of the hand, Yap et al. reported that three cases necessitated forearm amputation [15]. They emphasized the critical importance of prompt identification of the causative organism and the initiation of appropriate antimicrobial therapy, coupled with surgical intervention. They also recommended escalation to vancomycin in cases of methicillin-resistant *S. aureus* infection or failure to respond within 48 hours. Dadras et al. reported 22 cases of pyogenic wrist arthritis [16], 11 of which were associated with SL ligament repair or arthroscopic surgery. Seven of these cases improved with intra-articular lavage alone, whereas 15 required external fixation. Four patients underwent articular cartilage debridement, and 10 required carpectomy. Postoperative outcomes included an average DASH score of 34 points, an average ROM ratio of 49% compared to healthy controls, and an average grip strength ratio of 70%, highlighting the challenges in treating this condition. Our case shares several similarities with the Dadras report, including the presence of an MSSA infection following arthroscopic surgery for SL ligament injury and a treatment regimen involving intra-articular lavage and external fixation. At the final evaluation, the patient exhibited a ROM ratio of 40% and a grip strength ratio of 77%. Fortunately, despite these limitations, the patient reported minimal subjective complaints, with a DASH score of 6.25, and required only partial immobilization. Quadlbauer et al. reported a case of spontaneous radioscapholunate (RSL) fusion post-wrist infection caused by a dog bite [17]. In their case, RSL joint destruction also began two months after injury, and RSL was spontaneously fused after seven years. Our case is similar to theirs, and while it may appear to be an extreme idea, spontaneous radiocarpal fusion could be a natural progression after severe pyogenic arthritis of the wrist. As they stated, the clinical outcome of partial radial carpal fusion was relatively positive, suggesting that our treatment strategy was appropriate.

## Conclusions

This case highlights the importance of early intervention in suspected infections following percutaneous pinning. Prompt removal of the pin is crucial in preventing the progression of infection. If the infection advances to osteomyelitis or septic arthritis, comprehensive surgical debridement and the administration of targeted antimicrobial therapy are imperative for effective management.
